# Supplementary material for: The impact of pandemic-related worry on cognitive functioning and risk-taking
Source: PLoS One. 2021 Nov 18;16(11):e0260061. doi: 10.1371/journal.pone.0260061 (PMC8601558; doi:10.1371/journal.pone.0260061)
Supplement: S1 File — (DOCX) [file pone.0260061.s001.docx]

**Supplementary Materials**

**Exclusion Criteria**

Participants’ locations were determined via participant’s IP addresses. Of the 1517 total participants, data from 12 participants were discarded as their location was determined to be outside of the United States. Next, we removed the data of 58 participants who reported the following unlikely responses:

1. Having learned English at an age equal or greater than their reported age (22 participants)
2. The number of children or adults in their house was greater than 3 standard deviations from the mean across samples (36 participants)

Following this, we checked if any participants had previously participated in one or more of our previous experiments which were used as the pre-pandemic baseline sample. Consequently, we removed the data of 76 participants from the pandemic sample who had their unique Mturk “worker-ID” exactly match one of the participants from the pre-pandemic sample, leaving us with responses from 1371 participants on the four tasks.

Of the remaining 1371 participants, questionnaire responses (i.e., PSS, FSS, FCQ) of 72 participants (Wave 1: 18; Wave 2: 27; Wave 3: 27) failed to record, due to issues with the server.

To ensure the quality of our data, we further excluded participants on a task-by task basis based on their performance. Below we describe the exclusions made by task. Additionally, for the regressions where income and gender were added as covariates, data from 12 participants who we could not dummy code (i.e. opted not to report their income level 6 participants or did not identify as either female of male 6 participants).

***Digit Symbol-Coding Task***

In order to ensure that participants were sufficiently engaged, and understood the task, we excluded the data of 335 participants (Pre-pandemic: 10, Pandemic: 325) who did not achieve an accuracy of at least 70% in their last run.

***Task Switching***

In order to ensure participants understood the task, and were sufficiently motivated to complete it, we excluded the data from those participants who failed to respond to more than 10% (8) trials (pandemic sample: 119, pre-pandemic sample: 4 participants), and those who responded correctly to fewer than 70% of trials (pandemic sample: 584, pre-pandemic sample: 32 participants). We also excluded trials with exceptionally fast or slow response-times by removing trials 3 standard deviations away from the participant mean (0.23%).

***Dot Pattern Expectancy Task***

Following the previous tasks, we excluded the data of those who missed more than 10% (12) trials (Pre-pandemic: 12, Pandemic: 365) and those who achieved less than 70% accuracy (Pre-pandemic: 3, Pandemic: 198). Again, we removed trials with response-times greater than or less than 3 standard deviations away from the participant mean (1.1%).

***Risky Decision-Making Task***

Again, we excluded the data of those who failed to respond before the deadline on more than 20 of trials (Pre-pandemic: 11, Pandemic: 253), and those who failed to respond correctly to more than 70% of catch trials where the certain option was favored in terms of EV (Pre-pandemic: 25, Pandemic: 559). Mirroring the other tasks, we removed trials with response-times 3 standard deviations away from the participant mean (1.0%).

**Supplemental Results**

***FCQ as a function of exclusion status***

In order to ensure that our task-based exclusions did not disproportionately remove those reporting higher in pandemic-worry, we compared the FCQ scores between those excluded and those included on a task-by-task basis using Welch two sample t-tests. However, we failed to find a difference in pandemic-worry between those whose data we excluded and those included across all four tasks (DS: t(321.68) = -1.1811, p = 0.2384; TS: t(1275.1) = -0.63132, p = 0.5279; DPX: t (987.36) = -0.17759, p = 0.8591; RDM: t(306.51)= -0.61602, p=0.5383). Together, these results suggest that the exclusion criteria used here did not covary with our predictor of interest.

***Risky decision-making RTs and catch accuracy***

To ensure any changes in risk preferences were not due to a change in decision strategy or an increase in random responding, we analyzed task RTs and catch trial responses. Critically, comparing the pre-pandemic sample to the pandemic sample, we did not find any evidence that participants were faster in deciding ($\beta$ = -0.0764, CI = [-0.1676, 0.0149], p =.101), nor were they any worse at responding to catch trials where the expected value greatly favored the certain gain ($\beta$ =-0.0550, CI = [-0.3144 – 0.2043], p = .677), suggesting that the changes in risk preferences were not due to more impulsive deciding. Nor did we find that catch trial accuracy varied between waves (Wave 2: $\beta$ =0.0083, CI = [-0.1806 – 0.1971], p = .931; Wave 3: $\beta$ =-0.1292, CI = [-0.3246 – 0.0662], p = .195; linear effect: $\beta$ =-0.0616, CI = [-0.15986 – 0.0366]).

Importantly, we also did not observe a relationship between FCQ scores and accuracy on catch trials which favored the certain option ($\beta$ = -0.0129, CI= [-0.0918 – 0.0660], p= .749) suggesting that decision-making strategies (i.e., using EV) largely remained consistent. Furthermore, FCQ scores were also not significantly related to faster decisions ($\beta$ = -0.0215, CI =[-0.0440, 0.0010], *p = .061*), suggesting that the behavioural changes were not due to less cautious decision-making.

**Table S1** *Results of linear regression comparing digit-symbol score (total correct responses in 90 seconds) between the waves of the pandemic sample (dummy coded; baseline =Wave 1) and z-scored questionnaire scores, controlling for age (mean centered), gender (male = -0.5, female =0.5), COVID-19 symptoms (symptoms = 0.5, no symptoms =-0.5), risky for contracting COVID-19 (elevated =0.5, not elevated =-0.5), and total number of tasks the participant had completed during the experimental session (0, 1, 2 or 3). Linear regressors for income, education level and years on Mturk were included in the model.*

| *Predictors* | *Estimates* | *CI* | *p* |
| --- | --- | --- | --- |
| Intercept | 44.4053 | 41.6827 – 47.1279 | <0.001* |
| COVID -19 symptoms | -1.7712 | -3.8997 – 0.3573 | 0.103 |
| Wave 2 (vs Wave 1) | -2.2955 | -3.8630 – -0.7281 | 0.004* |
| Wave 3 (vs Wave 1) | -2.1246 | -3.8492 – -0.4000 | 0.0168* |
| Total tasks complete | 0.7761 | 0.2152 – 1.3371 | 0.007* |
| Age (mean centered) | -0.3251 | -0.3882 – -0.2620 | <0.001* |
| Gender (male vs female) | 1.2264 | -0.1382 – 2.5911 | 0.078 |
| Income | 0.7932 | 0.3221 – 1.2644 | 0.001* |
| Years on Mturk | 1.2119 | 0.7784 – 1.6453 | <0.001* |
| Highest education level | -0.8549 | -1.4616 – -0.2482 | 0.006* |
| Risk for strong COVID-19 | -1.4867 | -3.1321 – 0.1588 | 0.077* |
| Adults living in house | 0.1141 | -0.4346 – 0.6629 | 0.683 |
| Children living in house | -1.4285 | -1.9388 – -0.9182 | <0.001* |
| Focus (Z-scored) | 2.8831 | 1.8961 – 3.8702 | <0.001* |
| FSS (Z-scored) | -0.2526 | -0.9506 – 0.4454 | 0.478 |
| PSS (Z-scored) | -0.3610 | -1.0337 – 0.3118 | 0.293 |
| FCQ (Z-scored) | -0.9407 | -1.5918 – -0.2897 | 0.005* |

**Table S2** *Results of linear mixed-effects regression comparing correct digit-symbol log RT between the waves of the pandemic sample (dummy coded; baseline =Wave 1) and z-scored questionnaire scores and correct responses (correct=0.5, incorrect=-0.5), controlling for age (mean centered), gender (male = -0.5, female =0.5), COVID-19 symptoms (symptoms = 0.5, no symptoms =-0.5), risky for contracting COVID-19 (elevated =0.5, not elevated =-0.5), and total number of tasks the participant had completed during the experimental session (0, 1, 2 or 3). Linear regressors for income, education level and years on Mturk were included in the model.*

| *Predictors* | *Estimates* | *CI* | *p* |
| --- | --- | --- | --- |
| Intercept | 7.4714 | 7.4120 – 7.5308 | <0.001* |
| COVID -19 symptoms | 0.0434 | -0.0031 – 0.0900 | 0.067 |
| Wave 2 (vs Wave 1) | 0.0424 | 0.0082 – 0.0765 | 0.015* |
| Wave 3 (vs Wave 1) | 0.0538 | 0.0161 – 0.0914 | 0.005* |
| Total tasks complete | -0.0166 | -0.0288 – -0.0043 | 0.00z |
| Age (mean centered) | 0.0072 | 0.0059 – 0.0086 | <0.001 |
| Gender (male vs female) | -0.0270 | -0.0568 – 0.0028 | 0.076 |
| Income | -0.0188 | -0.0291 – -0.0085 | <0.001 |
| Years on Mturk | -0.0281 | -0.0376 – -0.0187 | <0.001 |
| Highest education level | 0.0151 | 0.0018 – 0.0283 | 0.026 |
| Risk for strong COVID-19 | 0.0362 | 0.0003 – 0.0722 | 0.048 |
| Adults living in house | -0.0028 | -0.0148 – 0.0092 | 0.645 |
| Children living in house | 0.0211 | 0.0099 – 0.0323 | <0.001 |
| Focus (Z-scored) | -0.0507 | -0.0723 – -0.0292 | <0.001 |
| FSS (Z-scored) | -0.0002 | -0.0155 – 0.0150 | 0.976 |
| PSS (Z-scored) | 0.0070 | -0.0076 – 0.0217 | 0.346 |
| FCQ (Z-scored) | 0.0190 | 0.0048 – 0.0332 | 0.009 |

***Table S3*** *Table containing dates of data collection and sample sizes for the pre-pandemic samples used in the study for comparison to the pandemic sample.*

| Task | Date | N |
| --- | --- | --- |
| Digit Symbol Coding | June 12^th^ 2019 | 100 |
| Digit Symbol Coding | August 2^nd^ – 8^th^ 2018 | 100 |
| DPX | June 28^th^ – July 21^st^ 2013 | 99 |
| Task Switching | May 3^rd^ – July 23^rd^ 2019 | 193 |
| RDM | June 12^th^ 2019 | 100 |

**Table S4** *Results of linear mixed-effects regression comparing task switching log RT on the task-switching paradigm between the waves of the pandemic sample (dummy coded; baseline =Wave 1) and z-scored questionnaire scores, controlling for age (mean centered), gender (male = -0.5, female =0.5), COVID-19 symptoms (symptoms = 0.5, no symptoms =-0.5), risky for contracting COVID-19 (elevated =0.5, not elevated =-0.5), and total number of tasks the participant had completed during the experimental session (0, 1, 2 or 3). Linear regressors for income, education level and years on Mturk were included in the model.*

| *Predictors* | *Estimates* | *CI* | *p* |
| --- | --- | --- | --- |
| Intercept | 6.5837 | 6.5251 – 6.6423 | <0.001* |
| COVID-19 Symptoms | 0.0235 | -0.0266 – 0.0736 | 0.358 |
| Switch (vs Repeat) | 0.1037 | 0.0930 – 0.1144 | <0.001* |
| Wave 2 (vs Wave 1) | 0.0231 | -0.0086 – 0.0547 | 0.153 |
| Wave 3 (vs Wave 1) | 0.0366 | -0.0000 – 0.0732 | 0.050 |
| Response congruency | -0.0648 | -0.0702 – -0.0593 | <0.001* |
| Income | -0.0170 | -0.0268 – -0.0072 | 0.001* |
| Focus (Z-scored) | -0.0137 | -0.0377 – 0.0103 | 0.262 |
| Years on Mturk | -0.0125 | -0.0222 – -0.0029 | 0.011* |
| PSS (Z-scored) | 0.0061 | -0.0068 – 0.0190 | 0.357 |
| FSS (Z-scored) | -0.0047 | -0.0190 – 0.0097 | 0.525 |
| Adults in house | 0.0149 | 0.0009 – 0.0288 | 0.037* |
| FCQ (Z-scored) | -0.0005 | -0.0142 – 0.0132 | 0.946 |
| Children in House | 0.0065 | -0.0064 – 0.0193 | 0.323 |
| Age (mean centred) | 0.0024 | 0.0011 – 0.0037 | <0.001* |
| Risky for strong COVID-19 | 0.0098 | -0.0270 – 0.0467 | 0.601 |
| Highest level of Education | 0.0183 | 0.0060 – 0.0305 | 0.003* |
| Gender | -0.0280 | -0.0568 – 0.0008 | 0.057 |
| Total tasks completed | -0.0084 | -0.0205 – 0.0037 | 0.175 |
| Switch * Wave 2 | -0.0083 | -0.0236 – 0.0070 | 0.290 |
| Switch * Wave 3 | -0.0119 | -0.0293 – 0.0054 | 0.177 |
| Switch * PSS (Z-scored) | 0.0022 | -0.0040 – 0.0084 | 0.484 |
| Switch * FSS (Z-scored) | -0.0024 | -0.0092 – 0.0045 | 0.498 |
| Switch * FCQ (Z-scored) | -0.0015 | -0.0081 – 0.0050 | 0.647 |

**Table S5** *Results of logistic mixed-effects regression comparing correct responses (correct=1 incorrect =0) on the task-switching paradigm between the waves of the pandemic sample (dummy coded; baseline =Wave 1) and z-scored questionnaire scores, controlling for age (mean centered), gender (male = -0.5, female =0.5), COVID-19 symptoms (symptoms = 0.5, no symptoms =-0.5), risky for contracting COVID-19 (elevated =0.5, not elevated =-0.5), and total number of tasks the participant had completed during the experimental session (0, 1, 2 or 3). Linear regressors for income, education level and years on Mturk were included in the model.*

| *Predictors* | *LogOdds* | *CI* | *p* |
| --- | --- | --- | --- |
| Intercept | 1.9299 | 1.7250 – 2.1348 | <0.001* |
| COVID-19 symptoms | -0.1037 | -0.2759 – 0.0685 | 0.238 |
| Switch (vs. repeat) | -0.5719 | -0.6531 – -0.4907 | <0.001 |
| Wave 2 (vs wave 1) | -0.1199 | -0.2312 – -0.0085 | 0.035* |
| Wave 3 (vs wave 1) | -0.1363 | -0.2646 – -0.0081 | 0.037* |
| Response congruency (congruent vs incongruent) | 1.5571 | 1.4782 – 1.6359 | <0.001* |
| Income | 0.0106 | -0.0237 – 0.0449 | 0.544 |
| Focus (Z-scored) | 0.1716 | 0.0892 – 0.2540 | <0.001* |
| Years on Mturk | 0.0366 | 0.0032 – 0.0700 | 0.032* |
| FSS (Z-scored) | -0.0826 | -0.1325 – -0.0326 | 0.001* |
| Adults in house | 0.0005 | -0.0478 – 0.0488 | 0.984 |
| FCQ (Z-scored) | -0.0385 | -0.0865 – 0.0096 | 0.117 |
| Children in house | -0.0582 | -0.1025 – -0.0138 | 0.010* |
| Age (mean centred) | 0.0008 | -0.0038 – 0.0054 | 0.732 |
| Risk for strong COVID-19 | 0.0149 | -0.1129 – 0.1428 | 0.819 |
| Highest level of education | -0.0298 | -0.0728 – 0.0132 | 0.175 |
| Gender (male vs female) | 0.0767 | -0.0240 – 0.1773 | 0.135 |
| Total tasks completed | 0.0119 | -0.0305 – 0.0543 | 0.582 |
| PSS (Z-scored) | -0.0025 | -0.0476 – 0.0427 | 0.915 |
| Switch * Wave 2 | -0.0585 | -0.1725 – 0.0554 | 0.314 |
| Switch * Wave 3 | 0.0230 | -0.1036 – 0.1496 | 0.721 |
| Switch * FCQ (Z-scored) | 0.0066 | -0.0415 – 0.0547 | 0.788 |

**Table S6** *Results of mixed-effects logistic regression comparing correct responses (correct=1 incorrect =0) on the task-switching paradigm between the pre-pandemic sample and the collapsed pandemic sample (pre-pandemic= -0.5, pandemic = 0.5) controlling for age (mean centered), gender (male = -0.5, female =0.5) and total number of tasks the participant had completed during the experimental session (0, 1, 2 or 3)*

| *Predictors* | *LogOdds* | *CI* | *p* |
| --- | --- | --- | --- |
| Intercept | 2.1943 | 2.0799 – 2.3086 | <0.001 |
| Switch (vs. repeat) | -0.6539 | -0.7668 – -0.5410 | <0.001* |
| Study (pre-pandemic vs. pandemic) | -0.2443 | -0.3835 – -0.1051 | 0.001* |
| Response congruency (congruent vs. incongruent) | 1.5713 | 1.4996 – 1.6429 | <0.001 |
| Age (mean centred) | 0.0024 | -0.0013 – 0.0062 | 0.204 |
| Gender (male vs female) | 0.1111 | 0.0182 – 0.2040 | 0.019 |
| Total tasks complete | 0.0205 | -0.0235 – 0.0645 | 0.362 |
| Switch * Study | 0.0643 | -0.0578 – 0.1865 | 0.302 |

**Table S7** *Results of mixed-effects linear regression comparing log-transformed response-times on the task-switching paradigm between the pre-pandemic sample and the collapsed pandemic sample (pre-pandemic= -0.5, pandemic = 0.5) controlling for age (mean centered), gender (male = -0.5, female =0.5) and total number of tasks the participant had completed during the experimental session (0, 1, 2 or 3)*

| *Predictors* | *Estimates* | *CI* | *p* |
| --- | --- | --- | --- |
| Intercept | 6.5859 | 6.5560 – 6.6158 | <0.001* |
| Switch (vs. repeat) | 0.1016 | 0.0875 – 0.1158 | <0.001* |
| Study (pre-pandemic vs. pandemic) | -0.0066 | -0.0437 – 0.0304 | 0.726 |
| Response congruency (congruent vs. incongruent) | -0.0631 | -0.0679 – -0.0583 | <0.001* |
| Age (mean centred) | 0.0018 | 0.0008 – 0.0028 | 0.001* |
| Gender (male vs female) | -0.0351 | -0.0602 – -0.0101 | 0.006* |
| Total tasks complete | -0.0082 | -0.0201 – 0.0037 | 0.176 |
| Switch * Study | -0.0045 | -0.0199 – 0.0110 | 0.572 |

***Table S8*** *Average accuracy and median RTs for the four trial types in the DPX task.*

| Stimulus | Median RT (SD) | Mean Accuracy (SD) |
| --- | --- | --- |
| AX | 562.0 (212.46) | 0.97 (0.03) |
| AY | 677.5 (209.43) | 0.82 (0.19) |
| BX | 485.0 (259.86) | 0.84 (0.23) |
| BY | 512.0 (265.50) | 0.95 (0.12) |

**Table S9** *Results of linear regression comparing proactive behavioural index (PBI) between the waves of the pandemic sample (dummy coded; baseline =Wave 1) and z-scored questionnaire scores, controlling for age (mean centered), gender (male = -0.5, female =0.5), COVID-19 symptoms (symptoms = 0.5, no symptoms =-0.5), risky for contracting COVID-19 (elevated =0.5, not elevated =-0.5), and total number of tasks the participant had completed during the experimental session (0, 1, 2 or 3). Linear regressors for income, education level and years on Mturk were included in the model.*

| *Predictors* | *Estimates* | *CI* | *p* |
| --- | --- | --- | --- |
| Intercept | 0.2373 | 0.1756 – 0.2989 | <0.001 |
| COVID-19 symptoms | -0.0168 | -0.0523 – 0.0187 | 0.353 |
| Wave 2 (vs. Wave1) | 0.0015 | -0.0214 – 0.0244 | 0.899 |
| Wave 3 (vs. Wave 1) | -0.0170 | -0.0432 – 0.0092 | 0.202 |
| Total tasks complete | 0.0048 | -0.0041 – 0.0137 | 0.286 |
| Age (mean centred) | -0.0009 | -0.0018 – 0.0001 | 0.069 |
| Gender (male vs female) | 0.0036 | -0.0170 – 0.0242 | 0.732 |
| Income | -0.0008 | -0.0078 – 0.0062 | 0.821 |
| Years on Mturk | 0.0043 | -0.0027 – 0.0112 | 0.230 |
| Highest level of education | 0.0001 | -0.0088 – 0.0090 | 0.982 |
| Risk for strong COVID-19 | 0.0003 | -0.0258 – 0.0264 | 0.982 |
| Adults in house | -0.0022 | -0.0113 – 0.0070 | 0.642 |
| Children in house | 0.0046 | -0.0038 – 0.0131 | 0.284 |
| Focus (Z-scored) | 0.0035 | -0.0136 – 0.0205 | 0.690 |
| FSS (Z-scored) | 0.0083 | -0.0019 – 0.0186 | 0.112 |
| PSS (Z-scored) | -0.0108 | -0.0200 – -0.0015 | 0.023* |
| FCQ (Z-scored) | -0.0020 | -0.0034 – -0.0005 | 0.010* |

**Table S10** *Results of logistic regression predicting correct responses on the DPX as a function of the waves of the pandemic sample (dummy coded; baseline =Wave 1) and z-scored questionnaire scores, controlling for age (mean centered), gender (male = -0.5, female =0.5), COVID-19 symptoms (symptoms = 0.5, no symptoms =-0.5), risky for contracting COVID-19 (elevated =0.5, not elevated =-0.5), and total number of tasks the participant had completed during the experimental session (0, 1, 2 or 3). Linear regressors for income, education level and years on Mturk were included in the model.*

| *Predictors* | *Log Odds* | *CI* | *p* |
| --- | --- | --- | --- |
| Intercept | 3.0750 | 2.6303 – 3.5197 | <0.001 |
| COVID-19 symptoms | -0.2635 | -0.5130 – -0.0140 | 0.038 |
| Wave 2 (vs. Wave1) | 0.0577 | -0.1104 – 0.2257 | 0.501 |
| Wave 3 (vs. Wave 1) | -0.0084 | -0.1981 – 0.1812 | 0.930 |
| Total tasks complete | -0.0533 | -0.1177 – 0.0111 | 0.105 |
| Age (mean centred) | 0.0097 | 0.0027 – 0.0167 | 0.006 |
| Gender (male vs female) | 0.1301 | -0.0213 – 0.2815 | 0.092 |
| Income | 0.0535 | 0.0023 – 0.1047 | 0.041 |
| Years on Mturk | 0.0377 | -0.0124 – 0.0879 | 0.140 |
| Highest level of education | -0.0426 | -0.1077 – 0.0225 | 0.199 |
| Risk for strong COVID-19 | -0.3045 | -0.4899 – -0.1190 | 0.001 |
| Adults in house | -0.0276 | -0.0907 – 0.0355 | 0.392 |
| Children in house | -0.1811 | -0.2414 – -0.1207 | <0.001 |
| Focus (Z-scored) | 0.3599 | 0.2416 – 0.4781 | <0.001 |
| FSS (Z-scored) | -0.0311 | -0.1063 – 0.0441 | 0.417 |
| PSS (Z-scored) | -0.0700 | -0.1393 – -0.0007 | 0.048 |
| FCQ (Z-scored) | -0.0062 | -0.0170 – 0.0046 | 0.261 |

**Table S11** *Table containing the stimuli used in the risky decision-making task to assess risk preferences. Every row represents a set of two stimuli, one in the gain frame and one in the loss frame. Stimuli in the loss frame are obtained by subtracting a fixed amount from the outcomes of the gain prospects. For the risky option, the remaining outcome probability (i.e. 1-P(win)) reflects the chance of winning $0.*

| Gain P(Win) | Gain Risky | Gain Certain | Loss P(Win) | Loss Risky | Loss Certain | ΔEV  Gain | ΔEV  Loss |
| --- | --- | --- | --- | --- | --- | --- | --- |
| 0.99 | 100 | 99 | 0.01 | -100 | -1 | 0 | 0 |
| 0.99 | 200 | 198 | 0.01 | -200 | -2 | 0 | 0 |
| 0.95 | 200 | 190 | 0.05 | -200 | -10 | 0 | 0 |
| 0.95 | 100 | 95 | 0.05 | -100 | -5 | 0 | 0 |
| 0.9 | 200 | 180 | 0.1 | -200 | -20 | 0 | 0 |
| 0.9 | 100 | 90 | 0.1 | -100 | -10 | 0 | 0 |
| 0.1 | 100 | 10 | 0.9 | -100 | -90 | 0 | 0 |
| 0.1 | 200 | 20 | 0.9 | -100 | -90 | 0 | 0 |
| 0.05 | 200 | 10 | 0.95 | -200 | -190 | 0 | 0 |
| 0.05 | 100 | 5 | 0.95 | -100 | -95 | 0 | 0 |
| 0.01 | 100 | 1 | 0.99 | -100 | -99 | 0 | 0 |
| 0.01 | 200 | 2 | 0.99 | -200 | -198 | 0 | 0 |

**Table S12** *Results of mixed-effects logistic regression risky choice (risky =1, certain =0) between the waves of the pandemic sample (dummy coded; baseline =Wave 1) and z-scored questionnaire scores, controlling for age (mean centered), gender (male = -0.5, female =0.5), COVID-19 symptoms (symptoms = 0.5, no symptoms =-0.5), risky for contracting COVID-19 (elevated =0.5, not elevated =-0.5), and total number of tasks the participant had completed during the experimental session (0, 1, 2 or 3). Linear regressors for income, education level and years on Mturk were included in the model.*

| *Predictors* | *Log-Odds* | *CI* | *p* |
| --- | --- | --- | --- |
| Intercept | -0.6718 | -0.9974 – -0.3461 | <0.001* |
| COVID-19 symptoms | 0.0208 | -0.2502 – 0.2918 | 0.881 |
| Wave 2 (vs wave 1) | -0.0736 | -0.2595 – 0.1122 | 0.438 |
| Wave 3 (vs wave 1) | -0.1974 | -0.4053 – 0.0106 | 0.063 |
| Total tasks completed | 0.0254 | -0.0437 – 0.0944 | 0.472 |
| Age (mean centred) | -0.0060 | -0.0141 – 0.0020 | 0.143 |
| Gender (male vs female) | 0.1693 | 0.0016 – 0.3370 | 0.048* |
| Income | 0.0293 | -0.0279 – 0.0865 | 0.316 |
| Years on Mturk | 0.0769 | 0.0223 – 0.1314 | 0.006* |
| Highest level of education | -0.0890 | -0.1599 – -0.0181 | 0.014* |
| Risk for strong COVID-19 | -0.1459 | -0.3497 – 0.0579 | 0.160 |
| Adults in house | 0.0210 | -0.0467 – 0.0887 | 0.543 |
| Children in house | -0.0919 | -0.1555 – -0.0283 | 0.005* |
| Frame (losses vs gains) | -1.3200 | -1.4430 – -1.1969 | <0.001* |
| Prob (likely vs unlikely) | -0.0673 | -0.1424 – 0.0077 | 0.079 |
| FCQ (Z-scored) | -0.0450 | -0.1250 – 0.0350 | 0.270 |
| PSS (Z-scored) | 0.0242 | -0.0529 – 0.1013 | 0.538 |
| FSS (Z-scored) | 0.0190 | -0.0629 – 0.1008 | 0.650 |
| Focus (Z-scored) | 0.1412 | 0.0136 – 0.2689 | 0.030* |
| Frame * Prob | 0.2560 | 0.1744 – 0.3375 | <0.001* |
| FCQ * Frame | 0.0226 | -0.0977 – 0.1429 | 0.713 |
| FCQ * Prob | -0.0281 | -0.1013 – 0.0452 | 0.452 |
| FCQ * Frame * Prob | 0.1720 | 0.0929 – 0.2511 | <0.001* |

**Table S13** *Results of mixed-effect logistic regression comparing risky choice (risky=1, certain=0) between the pre-pandemic sample and the collapsed pandemic sample (pre-pandemic= -0.5, pandemic = 0.5) controlling for age (mean centered), gender (male = -0.5, female =0.5) and total number of tasks the participant had completed during the experimental session (0, 1, 2 or 3)*

| *Predictors* | *LogOdds* | *CI* | *p* |
| --- | --- | --- | --- |
| Intercept | -0.4564 | -0.7185 – -0.1942 | 0.001* |
| Frame (loss vs gains) | -2.0629 | -2.4669 – -1.6589 | <0.001* |
| Prob (likely vs unlikely) | -0.3408 | -0.5825 – -0.0990 | 0.006* |
| Study (pre-pandemic vs pandemic) | -0.3490 | -0.6417 – -0.0563 | 0.019* |
| Total tasks complete | 0.0447 | -0.0267 – 0.1161 | 0.220 |
| Age (mean centred) | -0.0059 | -0.0137 – 0.0019 | 0.141 |
| Gender (male vs female) | 0.2691 | 0.1092 – 0.4290 | 0.001* |
| Frame * Prob | -0.2287 | -0.4967 – 0.0393 | 0.094 |
| Study * Frame | 0.7465 | 0.3242 – 1.1688 | 0.001* |
| Study * Prob | 0.2571 | 0.0043 – 0.5100 | 0.046* |
| Study * Frame * Prob | 0.4723 | 0.1925 – 0.7522 | 0.001* |

**Table S14** *Results of mixed-effects logistic regression risky choice (risky =1, certain =0) between the waves of the pandemic sample (dummy coded; baseline =Wave 1) and z-scored questionnaire scores, controlling for age (mean centered), gender (male = -0.5, female =0.5), COVID-19 symptoms (symptoms = 0.5, no symptoms =-0.5), risky for contracting COVID-19 (elevated =0.5, not elevated =-0.5), and total number of tasks the participant had completed during the experimental session (0, 1, 2 or 3). Linear regressors for income, education level and years on Mturk were included in the model.*

| *Predictors* | *Log-Odds* | *CI* | *p* |
| --- | --- | --- | --- |
| (Intercept) | -0.6692 | -0.9955 – -0.3430 | <0.001* |
| COVID-19 symptoms | 0.0307 | -0.2412 – 0.3025 | 0.825 |
| Wave 2 (vs Wave 1) | -0.0804 | -0.2665 – 0.1056 | 0.397 |
| Wave 3 (vs Wave 2) | -0.2011 | -0.4094 – 0.0072 | 0.058 |
| Total tasks completed | 0.0266 | -0.0425 – 0.0957 | 0.450 |
| Age (mean centred) | -0.0059 | -0.0139 – 0.0022 | 0.152 |
| Gender (male vs female) | 0.1644 | -0.0035 – 0.3323 | 0.055 |
| Income | 0.0295 | -0.0277 – 0.0868 | 0.312 |
| Years on Mturk | 0.0774 | 0.0228 – 0.1320 | 0.005* |
| Highest level of education | -0.0902 | -0.1611 – -0.0192 | 0.013* |
| Risk for strong COVID-19 | -0.1542 | -0.3583 – 0.0500 | 0.139 |
| Adults in house | 0.0209 | -0.0469 – 0.0887 | 0.546 |
| Children in house | -0.0924 | -0.1560 – -0.0288 | 0.004* |
| PSS (Z-Scored) | 0.0213 | -0.0558 – 0.0985 | 0.588 |
| FSS (Z-Scored) | 0.0204 | -0.0615 – 0.1024 | 0.625 |
| Focus (Z-Scored) | 0.1420 | 0.0141 – 0.2699 | 0.030* |
| FCQ (Z-Scored) | -0.0470 | -0.1270 – 0.0331 | 0.250 |
| Frame (losses vs gains) | -1.4005 | -1.5968 – -1.2041 | <0.001* |
| Prob (likely vs unlikely) | 0.0632 | -0.0558 – 0.1822 | 0.298 |
| Frame * Prob | 0.1087 | -0.0206 – 0.2379 | 0.099 |
| Wave 2 * Frame | -0.1169 | -0.4056 – 0.1719 | 0.428 |
| Wave 2 * Prob | -0.1984 | -0.3737 – -0.0230 | 0.027* |
| Wave 3 * Frame | 0.4281 | 0.1256 – 0.7306 | 0.006* |
| Wave 3 * Prob | -0.2422 | -0.4275 – -0.0570 | 0.010* |
| Wave 2 * Frame * Prob | -0.0627 | -0.2541 – 0.1286 | 0.521 |
| Wave 3 * Frame * Prob | 0.6322 | 0.4278 – 0.8366 | <0.001* |

**Table S15** *Table containing the frequency of self-reported gender by wave, including X^2^ statistic reflecting the comparison of response between waves and associated p-value.*

|  |  | Wave 1 | Wave 2 | Wave 3 | statistic | p |
| --- | --- | --- | --- | --- | --- | --- |
| Gender | Female | 172 | 166 | 138 | X^2^(6)=20.72 | 0.002 |
|  | Male | 275 | 287 | 327 |  |  |
|  | Other | 4 | 2 | 0 |  |  |

**Table S16** *Table containing the frequency of responses to demographic questions pertaining to income, education, and experience on Mturk by wave. Additionally, X^2^ statistics reflecting the comparison of response between waves and associated p-values are included.*

|  |  | Wave 1 | Wave 2 | Wave 3 | X^2^ statistic | p |
| --- | --- | --- | --- | --- | --- | --- |
| Household income | $25K or less | 51 | 51 | 39 | X^2^(10)=20.716 | .023 |
|  | $25K - $35K | 62 | 62 | 64 |  |  |
|  | $35K - $50K | 91 | 83 | 123 |  |  |
|  | $50K - $75K | 133 | 140 | 158 |  |  |
|  | $75K - $100K | 65 | 71 | 53 |  |  |
|  | $100K or more | 46 | 45 | 28 |  |  |
| Education | Associate’s Degree | 45 | 37 | 26 | X^2^(10)=74.187 | <.001 |
|  | Bachelor’s | 222 | 245 | 268 |  |  |
|  | Postgraduate degree | 65 | 77 | 126 |  |  |
|  | High School | 41 | 31 | 20 |  |  |
|  | Some College no degree | 78 | 61 | 25 |  |  |
|  | Some High School | 0 | 4 | 0 |  |  |
| Years on Mturk | Less than a week | 6 | 7 | 13 | X^2^(12)=89.086 | <.001 |
|  | A few weeks | 16 | 20 | 27 |  |  |
|  | One month | 22 | 29 | 68 |  |  |
|  | Several Months | 93 | 113 | 101 |  |  |
|  | One year | 46 | 47 | 93 |  |  |
|  | A little over a year | 72 | 61 | 50 |  |  |
|  | More than 2 years | 196 | 178 | 113 |  |  |

**Table S17.** *Pearson correlation between performance on executive functioning tasks, using listwise deletion for those performing below criterion across all three waves of data collection. Digit symbol scores were computed as the total correct responses within 90 seconds of the Digit symbol coding task. Proactive behavioral index (PBI) was estimated as (RT_AY_ – RT_BX_)/(RT_AY_ + RT_BX_). Switch Costs computed as the empirical Bayes estimate of the person-level effect of task switches on log RTs.*

|  | *Switch Costs* | *Digit Symbol Score* | *PBI* |
| --- | --- | --- | --- |
| *Switch Costs* |  | *0.112** | *0.002* |
| *Digit Symbol Score* | *0.112** |  | *0.124** |
| *PBI* | *0.002* | *0.124** |  |
| *Computed correlation used pearson-method with listwise-deletion.*  ** p < .05* | | | |
